# Clinical spectrum and survival outcomes of malignancies in pediatric patients with inborn errors of immunity

**DOI:** 10.3389/fimmu.2026.1904173

**Published:** 2026-08-14

**Authors:** Nazli Deveci Demirbas, Zehra Sule Haskologlu, Tutku Baylan, Candan Islamoglu, Sevgi Kostel Bal, Kubra Baskin, Burcin Gul, Caner Aytekin, Gulsah Kaygusuz, Tanil Kendirli, Suat Fitoz, Sonay Incesoy Ozdemir, Handan Dincaslan, Isinsu Kuzu, Nurdan Tacyildiz, Emel Unal, Figen Dogu, Aydan Ikinciogullari

**Affiliations:** 1 Division of Pediatric Allergy and Immunology, Department of Pediatrics, Ankara University Faculty of Medicine, Ankara, Türkiye; 2 Department of Pediatric Allergy and Immunology, Dr. Sami Ulus Child Health and Diseases Training and Research Hospital, Ankara, Türkiye; 3 Department of Pathology, Ankara University Faculty of Medicine, Ankara, Türkiye; 4 Division of Pediatric Intensive Care, Department of Pediatrics, Ankara University Faculty of Medicine, Ankara, Türkiye; 5 Department of Radiology, Ankara University Faculty of Medicine, Ankara, Türkiye; 6 Division of Pediatric Hematology and Oncology, Department of Pediatrics, Ankara University Faculty of Medicine, Ankara, Türkiye

**Keywords:** hematopoietic stem cell transplantation, inborn errors of immunity, lymphoma, malignancy, pediatric oncology, survival

## Abstract

**Background:**

Inborn errors of immunity (IEI) predispose patients to malignancy, particularly lymphoma, but data on diagnostic sequence and outcomes are limited.

**Methods:**

We retrospectively analyzed IEI-associated malignancies diagnosed at a single center between January 2004 and December 2025.

**Results:**

Among 1,668 patients with IEI, 67 (4.0%) developed malignancy. Median ages at IEI and malignancy diagnosis were 8 and 10 years, respectively. Malignancy-first presentation occurred in 45 patients (67%), especially adolescents, and was associated with advanced-stage disease (p = 0.007). Lymphoid malignancies accounted for 90% of cases; non-Hodgkin lymphoma and Hodgkin lymphoma comprised 54% and 36%, respectively, and 84.7% presented with stage III-IV disease. Median event-free and overall survival were 66 and 155 months, respectively. In a clinically selected multivariable Cox model, NHL, solid/plasma cell tumors, older age at malignancy diagnosis, and DNA repair defect status were associated with worse OS. Twelve patients underwent allogeneic HSCT; one died early, and no post-transplant relapses occurred.

**Conclusion:**

Early immunologic evaluation and timely, individualized consideration of HSCT may support optimized management in children and adolescents, in whom cancer may be the first manifestation of IEI.

## Introduction

Inborn errors of immunity (IEI), formerly known as primary immunodeficiency disorders, comprise a heterogeneous group of genetic conditions, with more than 500 causative genes identified to date (1). These defects result in impaired development and/or function of various components of the immune system. Advances in next-generation sequencing have enabled high-throughput genomic analyses, substantially improving the understanding of the molecular and cellular mechanisms underlying IEI (2).

Individuals with IEI have a markedly increased risk of malignancy, especially hematologic cancers. The risk of lymphoma is estimated to be up to tenfold higher than in age- and sex-matched controls. In pediatric IEI cohorts, the overall risk of cancer ranges from 4% to 25%, with hematologic malignancies predominating (3–6). About two-thirds of these cancers are lymphomas, with non-Hodgkin lymphoma (NHL) being more frequent than Hodgkin lymphoma (HL) (4, 7).

Numerous mechanisms contribute to oncogenesis, including intrinsic molecular problems, impaired immunosurveillance, and chronic antigenic stimulation by infections or viruses (8). Epstein–Barr virus (EBV) has been implicated in 30% to 60% of lymphomas associated with IEI (9).

A recent extensive European Society for Immunodeficiencies (ESID) registry study, encompassing nearly 20,000 patients with IEI provided one of the most comprehensive epidemiological assessments of malignancy burden to date. In this cohort, 8.9% of patients developed cancer, and in a substantial proportion of patients, malignancy represented one of the first manifestations of the underlying immunodeficiency. B-cell NHL was the most common malignancy, whereas solid tumors and leukemias were less frequent. The study highlighted pronounced heterogeneity in cancer risk, age at onset, and malignancy spectrum across IEI subgroups, with earlier onset and higher intrinsic risk observed in combined immunodeficiencies, immune dysregulation disorders, and DNA repair defects (10).

Despite growing awareness of cancer predisposition in IEI, data on clinical presentation, the timing of malignancy relative to IEI diagnosis, and survival outcomes, particularly in pediatric and adolescent populations, remain limited. Single-center cohorts may provide complementary insights into disease behavior, treatment strategies, and long-term outcomes that are not fully captured in registry-based analyses. This study therefore aimed to delineate the spectrum, timing, and outcomes of malignancies in patients with IEI at a tertiary referral center, and to identify clinical factors associated with survival in this high-risk population.

## Methods

### Study design and setting

This retrospective single-center study was conducted at the Department of Pediatric Immunology, Ankara University School of Medicine. Medical records of patients with a confirmed diagnosis of IEI and malignancy between January 2004 and December 2025 were reviewed. Patients were identified through the institutional immunology registry, and oncologic data were obtained from medical records. The study was reviewed and approved by the Ankara University Clinical Research Ethics Committee Ankara University Human Research Ethics Committee (application no. 2024000184–3 [2024/184]; decision no. İ03-271-24; approval date: April 22, 2024). The study was conducted in accordance with the Declaration of Helsinki and relevant institutional and national requirements.

### Patient selection and data collection

Eligible patients fulfilled the 2019 European Society for Immunodeficiencies (ESID) Registry working definitions for IEI, and IEI entities/subgroups were classified according to the 2024 International Union of Immunological Societies (IUIS) classification. Individuals with secondary immunodeficiency were excluded (1, 11). Clinical, demographic, and laboratory data were extracted from electronic and paper-based medical records. Collected variables included age at IEI and malignancy diagnosis, sex, family history, parental consanguinity, history of recurrent infections, presence of bronchiectasis, lymphoproliferation, and other features of immune dysregulation. Immunologic parameters included serum immunoglobulin levels, isohemagglutinin responses, specific antibody responses, lymphocyte subsets, and EBV status, assessed in peripheral blood and/or tumor tissue. Ages at IEI and malignancy diagnosis were categorized into four groups (0–5, 6–11, 12–18, and >18 years) to allow for comparison of early-childhood, mid-childhood, adolescent, and adult-onset cases. Oncologic features included malignancy type, histologic subtype, disease stage, treatment modality, and hematopoietic stem cell transplantation (HSCT) outcomes.

### Classification and definitions

IEI subgroups were categorized according to the IUIS classification as combined immunodeficiency (CID), DNA repair defect, predominantly antibody deficiency (PAD), immune dysregulation (ID), and autoinflammatory disease (AID). Malignancies were classified as lymphoid (HL, NHL), solid tumor, and plasma cell tumor. Lymphoma subtypes were defined according to the 2022 World Health Organization fifth edition classification of hematolymphoid tumors and the 2022 International Consensus Classification, as applicable (12, 13). Lymphoma staging was performed using the Ann Arbor system.

### Laboratory analysis

Serum immunoglobulin concentrations were measured by nephelometry and interpreted according to age-adjusted reference ranges. Lymphocyte subsets were assessed by flow cytometry. EBV DNA was detected by polymerase chain reaction (PCR) in peripheral blood, and EBV status in tumor tissue was evaluated by *in situ* hybridization or immunohistochemistry when available.

### Genetic testing and variant interpretation

Because the cohort spans a 21-year period, genetic testing reflected the diagnostic approaches available at the time of evaluation and was not performed using a single uniform platform. Test selection was guided by the clinical and laboratory phenotype, preliminary diagnosis, family history, EBV status, and the genetic methods nationally available at the time. Patients underwent single-gene analysis, targeted EBV-focused next-generation sequencing (NGS) panel testing, fluorescence *in situ* hybridization (FISH), or whole-exome sequencing (WES).

Single-gene analysis was selected when the clinical phenotype strongly suggested a specific disorder or when a familial pathogenic variant had previously been identified. This group comprised 10 patients with suspected ataxia-telangiectasia, one with suspected Nijmegen breakage syndrome, and four relatives from families in which the molecular diagnosis had previously been established by WES. Targeted EBV-focused NGS panel testing was selected for patients with tissue and/or serum EBV positivity or a suspected EBV-driven lymphoproliferative phenotype when the findings did not indicate a single causative gene. These panels included the EBV-associated IEI genes available at the time of testing. WES was selected for patients with genetically heterogeneous, overlapping, or atypical phenotypes that could not be adequately evaluated using a single-gene or available targeted-panel approach and became the preferred broader testing strategy after 2019. FISH was used in one patient with a clinically suspected chromosomal abnormality. Because this was a retrospective cohort, these criteria guided test selection but did not constitute a fixed prospective algorithm; the final choice was also influenced by test availability at the time of evaluation.

Before 2019, genetic analyses were performed at different diagnostic centers. Consequently, exact panel composition, sequencing metrics, genome build, and transcript-level reporting were not uniformly available and could not be retrospectively harmonized. From 2019 onward, WES was increasingly performed using a standardized workflow. Genomic DNA was isolated from peripheral blood samples using a GeneAll DNA isolation kit. Exome libraries were prepared using the Illumina Nextera DNA Prep with Enrichment Kit, and sequencing was performed on the Illumina NextSeq 550 platform, generating 150-bp paired-end reads. Read mapping, variant calling, annotation, and copy-number variation (CNV) analysis were performed using SEQ Platform v8 (Genomize).

Quality metrics were available for WES performed from 2019 onward. The total number of reads ranged from 21.7 to 77.6 million (median, 46.1 million), and the mean depth of coverage ranged from 24.5× to 134.2× (median, 64.1×). The proportion of target regions, including exons and splice-site regions, covered at ≥20× ranged from 89.02% to 99.91%, whereas coverage at ≥50× ranged from 68.13% to 99.65%.

To identify potentially causative variants, exonic and splice-site variants were prioritized, synonymous variants were excluded, and rare variants with a minor allele frequency of <1% in population databases were evaluated. Filtering was performed stepwise, initially examining known IEI-associated genes, followed by phenotype-prioritized candidate genes and, when necessary, variants across the entire exome. Candidate variants were classified according to the American College of Medical Genetics and Genomics/Association for Molecular Pathology (ACMG/AMP) criteria. Identified variants were validated by Sanger sequencing when required, using standard protocols as previously described (14). The distribution of testing methods and diagnostic yield is summarized in **Supplementary Figures 1**, **2**.

### Statistical analysis

Statistical analyses were performed with IBM SPSS Statistics version 25. Categorical variables were expressed as numbers and percentages and compared using Pearson’s chi-square or Fisher’s exact tests, as appropriate. Continuous variables were summarized as medians with interquartile ranges and compared using Mann-Whitney U or Kruskal-Wallis tests. Because the AID category included only one patient, this case was retained only for descriptive completeness and was excluded from all comparative IEI subgroup analyses; subgroup comparisons were therefore restricted to CID, DNA repair defects, PAD, and ID. Exploratory comparisons of lymphocyte subsets were performed between HL and NHL. Solid and plasma cell tumors were not included in inferential comparisons because of their small and heterogeneous sample sizes. Continuous variables were compared using the Mann–Whitney U test, and p values were adjusted for multiple comparisons using the Benjamini–Hochberg procedure.

Overall survival (OS) was calculated from malignancy diagnosis to death from any cause or last follow-up, and event-free survival (EFS) to relapse, progression, secondary malignancy, death, or last follow-up. Survival curves were generated by the Kaplan-Meier method and compared with the log-rank test. A broad set of demographic, clinical, immunological, and malignancy-related variables was first examined in univariable analyses. Candidate variables for the multivariable Cox model were selected by considering univariable findings together with clinical relevance and previously reported prognostic importance; no fixed univariable p-value threshold was used as the sole criterion for model entry. Given the limited number of deaths (n = 26), the multivariable model was deliberately restricted to three clinically selected factors to reduce model instability and overfitting: malignancy type, age at malignancy diagnosis, and DNA repair defect status. Age at IEI diagnosis and age at malignancy diagnosis were strongly correlated and were therefore not entered simultaneously; age at malignancy diagnosis was selected because OS follow-up began at malignancy diagnosis. Treatment exposures were not included because they occurred after baseline and were susceptible to indication and selection bias. Plasma cell tumors were combined with solid tumors because of sparse events. The proportional hazards assumption was assessed using Schoenfeld residuals.

## Results

### Patient characteristics and distribution of IEI subtypes

Over a 21-year follow-up period at our center, malignancy was identified in 67 of 1,668 patients (4.0%) with IEI, excluding THI. By subgroup, malignancy occurred in 26.5% of patients with PAD, 17.7% with DNA repair defects, 15.2% with ID, 9.2% with CID/SCID, and 6.7% with AID. Among patients who developed malignancy, CID was the most frequent IEI category (n = 27), followed by ID (n = 15), PAD (n = 13), DNA repair defects (n = 11), and AID (n = 1). The distribution of underlying genetic defects within each IEI subgroup is summarized in **Table 1**.

**Table 1:** Malignancy distribution and representative genetic defects across IEI subgroups.

IEI subgroups	Example gene defects	HL, n (%)	NHL, n (%)	Solid, n (%)	Plasmacytoma, n (%)	Total, n (%)
CID	GIMAP5 (n=6) Artemis (n=2) DOCK8 (n=4) BCAP31 (n=1) IL2R def (n=1) ITK (n=1) STK4 (n=1) PIK3CD(n=1) 22q11.2 deletion (n=1) WAS (n=1) Undefined (n=8)	11(40.7%)	13 (48.1%)	1 (3.7%)	2 (7.4%)	27 (100%)
DNA repair defect	Ataxia-telangiectasia (n=10) Nijmegen (n=1)	2 (18.2%)	7 (63.6%)	2 (18.2%)	0	11 (100%)
PAD	NFKB1 (n=1) Undefined CVID (n=12)	5 (38.5%)	6 (46.1%)	2 (15.4%)	0	13 (100%)
ID	CD70 def (n=4) ALPS (n=2) CTLA4 (n=1) LRBA (n=1) CD137 (n=1) MAGT1 (n=1) RASGRP1 (n=1) TTP2 def (n=1) Undefined (n=3)	6 (40%)	9 (60%)	0	0	15 (100%)
AID	ADA2 (n=1)	0	1 (100%)	0	0	1 (100%)

AID, autoinflammatory disease; ALPS, autoimmune lymphoproliferative syndrome; CID, combined immunodeficiency; CVID, common variable immunodeficiency; HL, Hodgkin lymphoma; ID, immune dysregulation; NHL, non-Hodgkin lymphoma; PAD, predominantly antibody deficiency; WAS, Wiskott-Aldrich syndrome.

A targeted comparison included 13 patients with PAD and 53 with non-PAD IEI; the single patient with AID was excluded in accordance with the exploratory subgroup analysis. Median ages at malignancy diagnosis [11.0 years (IQR, 6.0–16.0) vs. 9.0 years (IQR, 5.0–13.0); unadjusted p = 0.283] and IEI diagnosis [12.0 years (IQR, 6.0–16.0) vs. 6.5 years (IQR, 3.9–13.0); unadjusted p = 0.072] did not differ significantly between the PAD and non-PAD groups. Malignancy type, lymphoma subtype, stage, rituximab exposure, radiotherapy, and allogeneic HSCT use were also comparable. EBV positivity was numerically lower in PAD (30.8% vs. 65.9%; unadjusted p = 0.051), but this difference was not significant after Benjamini–Hochberg correction (adjusted p = 0.335). Among patients with lymphoma, Burkitt lymphoma was more frequent in PAD (45.5% [5/11] vs. 14.6% [7/48]; unadjusted p = 0.036), although this *post-hoc* association did not remain significant after correction for subtype comparisons (adjusted p = 0.180). Hypogammaglobulinemia was more frequent in PAD (100% vs. 61.7%; adjusted p = 0.030), whereas recurrent infections were numerically less frequent (23.1% vs. 58.5%; adjusted p = 0.077).

Of the 67 patients, 41 (61.2%) were male and 26 (38.8%) were female (male to female ratio, 1.6:1). Sex distribution varied significantly among IEI subgroups (Fisher’s exact test, p = 0.001). All patients in the ID group were male, whereas other subgroups demonstrated a more balanced sex distribution (**Table 2**).

**Table 2:** Comparison of demographic, clinical, and laboratory features across IEI subgroups.

Characteristic	CID (n=27)	DNA repair defects (n=11)	PAD (n=13)	ID (n=15)	p, CID vs DNA repair	p, CID vs PAD	p, CID vs ID	p, DNA repair vs PAD	p, DNA repair vs ID	p, PAD vs ID
Sex (Male)	15 (56%)	5 (45%)	6 (46%)	15 (100%)	0.836	0.826	0.003	1.000	0.002	0.001
Recurrent infection	17 (63%)	9 (82%)	3 (23%)	5 (33%)	0.444	0.041	0.129	0.012	0.021	0.686
Bronchiectasis	10 (39%)	5 (50%)	3 (23%)	1 (7%)	0.801	0.477	0.061	0.221	0.050	0.326
Family history	13 (48%)	8 (73%)	4 (31%)	10 (67%)	0.282	0.333	0.405	0.100	1.000	0.128
Consanguinity	20 (74%)	11 (100%)	7 (54%)	8 (53%)	0.084	0.358	0.306	0.016	0.010	1.000
Autoimmunity	8 (30%)	2 (18%)	4 (31%)	4 (27%)	0.690	1.000	1.000	0.649	1.000	1.000
Lymphoproliferation	14 (52%)	2 (18%)	8 (62%)	9 (60%)	0.078	0.812	0.853	0.047	0.051	1.000
Median age at IEI diagnosis (range), years	12 (2 mo-25)	4 (2–7)	12 (3–39)	8 (4-14)	0.033	0.354	0.635	0.002	0.001	0.202
Median age at malignancy diagnosis (range), years	12 (4 mo-23)	9 (1-18)	11 (3-46)	6 (3-14)	0.079	1.000	0.017	0.191	0.566	0.049
EBV status	11 (48%)	3 (75%)	4 (31%)	13 (93%)	0.596	0.484	0.011	0.250	0.405	0.001

CID, combined immunodeficiency; DNA, deoxyribonucleic acid; EBV, Epstein-Barr virus; ID, immune dysregulation; IEI, inborn errors of immunity; mo, months; PAD, predominantly antibody deficiency.

### Age distribution and temporal sequence of diagnoses

The median age at IEI diagnosis was 8 years (IQR, 8; range, 2.5 months–39 years) and the median age at malignancy diagnosis was 10 years (IQR, 8; range, 4 months–46 years). The distribution of age groups at IEI diagnosis differed significantly across IEI subgroups (Fisher’s exact test, p = 0.006). DNA repair defects were predominantly diagnosed in early childhood (75% between 0 and 5 years), whereas CID showed a broader age distribution, frequently extending into adolescence. Predominantly antibody deficiencies were more commonly diagnosed in later childhood and adolescence, while ID disorders clustered mainly in the 6–11-year age group. In contrast, the distribution of age at malignancy diagnosis did not differ significantly among IEI subgroups (Fisher’s exact test, p = 0.256), although distinct patterns were observed.

**Figure 1:**
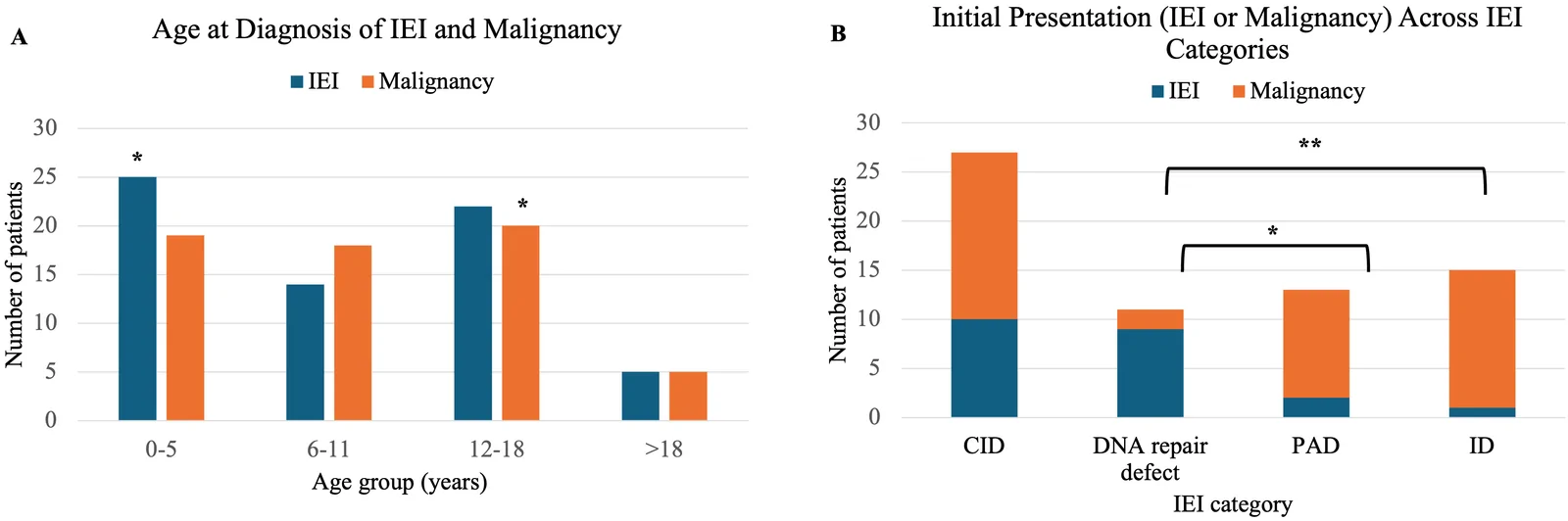
Age at diagnosis of IEI and malignancy and initial presentation patterns across immunodeficiency groups. **(A)** Distribution of patients across age groups at IEI and malignancy diagnosis (0–5, 6–11, 12–18, and >18 years). Asterisks highlight the predominance of IEI-first presentation in the 0–5-year group and malignancy-first presentation in the 12–18-year group. The overall association between age at IEI diagnosis and diagnostic sequence was significant (Fisher’s exact test, p < 0.001). **(B)** Initial presentation (IEI first or malignancy first) across IEI subgroups, excluding AID. Initial presentation differed significantly among groups (χ² (3) = 18.52, p < 0.001). Holm-adjusted *post-hoc* Fisher tests showed significant differences between DNA repair defects and PAD (*p = 0.015) and between DNA repair defects and ID (**p < 0.001), as indicated by brackets. Blue and orange bars represent IEI-first and malignancy-first presentation, respectively.

Malignancy preceded the diagnosis of IEI in 45 patients (67%), whereas 22 patients (33%) had been diagnosed with IEI before malignancy. A strong association was observed between age at IEI diagnosis and the temporal sequence of diagnoses (Fisher’s exact test, p < 0.001). IEI was more frequently diagnosed first in patients identified before 5 years of age, whereas malignancy was the initial diagnosis in most patients diagnosed at older ages, particularly adolescents (**Figure 1A**). The distribution of initial presentation (IEI vs malignancy) also differed significantly across IEI categories. Malignancy as the first diagnosis was most frequent in patients with ID (93.3%) and PAD (84.6%), whereas in patients with DNA repair defects, IEI was more commonly diagnosed before malignancy (81.8%) (Fisher’s exact test, p = 0.001) (**Figure 1B**).

### Diagnostic delay

The median diagnostic delay for IEI was 12 months (range, 0–200 months). Although patients with PAD and ID disorders exhibited numerically longer delays, no statistically significant difference was observed among IEI categories (Kruskal–Wallis test, H = 4.18, *p* = 0.382). Similarly, patients in whom malignancy preceded IEI diagnosis tended to have longer delays; however, this difference did not reach statistical significance (Mann–Whitney U = 433.5, *p* = 0.334).

### Genetic findings

Genetic testing was performed using single-gene analysis, targeted EBV-focused NGS panel testing, FISH, or WES according to clinical indication and test availability. A molecular/genetic diagnosis was established in 45 of 67 patients (67.2%). WES was the most frequently used method, performed in 42 patients and yielding a diagnosis in 24. Single-gene analysis was performed in 15 patients and confirmed the diagnosis in all. Targeted EBV-focused NGS panel testing was performed in nine patients and established a diagnosis in five. FISH was performed in one patient and resulted in a molecular diagnosis. Overall, 22 patients (32.8%) remained without a molecular diagnosis despite genetic evaluation.

### Characteristics of malignancies

**Figure 2:**
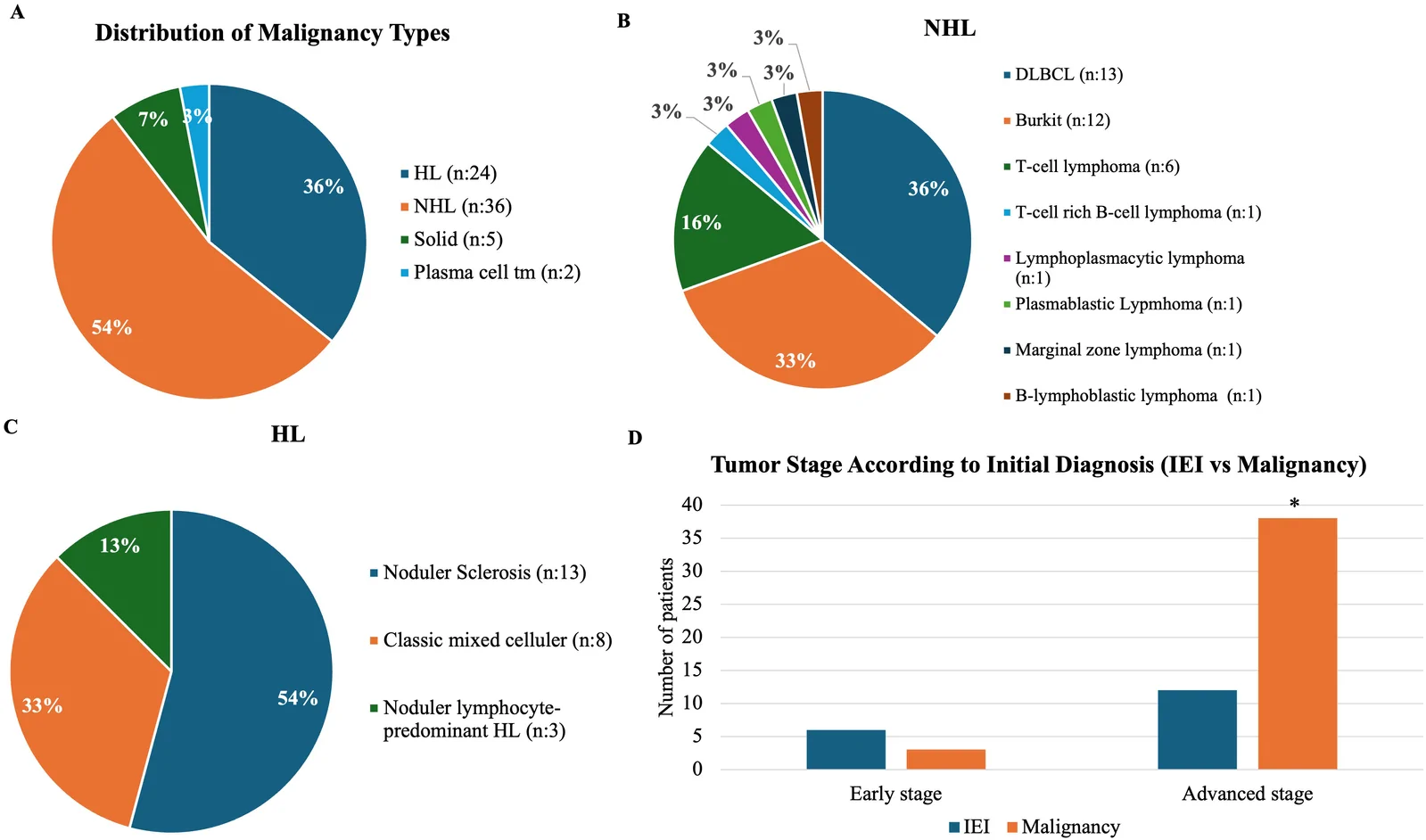
Distribution, histologic subtypes, and stage characteristics of malignancies in patients with inborn errors of immunity. **(A)** Overall distribution of malignancy types. **(B)** Histologic subtypes of non-Hodgkin lymphoma. **(C)** Histologic subtypes of Hodgkin lymphoma. **(D)** Tumor stage at malignancy diagnosis according to whether IEI or malignancy was diagnosed first. The asterisk indicates a statistically significant difference (*p* < 0.05). Abbreviations: DLBCL, diffuse large B-cell lymphoma; HL, Hodgkin lymphoma; IEI, inborn errors of immunity; NHL, non-Hodgkin lymphoma.

Lymphoid malignancies predominated, accounting for 90% of all cases. Non-Hodgkin lymphoma (NHL) was diagnosed in 36 patients (54%), followed by Hodgkin lymphoma (HL) in 24 patients (36%). Solid tumors were identified in five patients (7%), and plasma cell tumors in two patients (3%) (**Figure 2A**).

Among NHL cases, aggressive subtypes were most common and included diffuse large B-cell lymphoma (n = 13), Burkitt lymphoma (n = 12), T-cell lymphoma (n = 6), B-cell lymphoblastic lymphoma (n = 1), and plasmablastic lymphoma (n = 1), comprising 33 patients in total. Indolent lymphomas were rare and included marginal zone lymphoma (n = 1), lymphoplasmacytic lymphoma (n = 1), and T-cell–rich B-cell lymphoma (n = 1) (**Figure 2B**).

Among HL cases, nodular sclerosis classical HL was the most frequent subtype (54.2%), followed by mixed cellularity classical HL (33.3%) and nodular lymphocyte-predominant HL (12.5%) (**Figure 2C**).

At diagnosis, advanced-stage disease was common: 54.2% presented with stage IV disease, and 84.7% at stages III–IV. Early-stage disease (stage I–II) was observed in only 15.3% of patients. Disease stage did not differ significantly between HL and NHL (Fisher’s exact test, p = 0.845). In both groups, advanced-stage disease was predominant, with more than 80% of patients presenting with stage III–IV lymphoma. However, a significant association was observed between the temporal sequence of diagnoses and disease stage (Fisher’s exact test, p = 0.007). Patients in whom malignancy preceded IEI more frequently presented with advanced-stage disease (92.7% stage III–IV), compared with those diagnosed with IEI before malignancy (66.7%) (**Figure 2D**).

Nodal involvement was markedly more frequent in HL (79.2%) than in NHL (41.7%), whereas extranodal disease predominated in NHL (58.3%) (p = 0.004). Detailed information on biopsy sites and tumor types is provided in **Supplementary Table 1**.

### Clinical and immunological features

Bronchiectasis was identified in 29.7% of patients. A positive family history was reported in 52.2%, and parental consanguinity in 70.1%. Recurrent infections were documented in 50.7%, autoimmunity in 26.9%, and lymphoproliferative manifestations in 49.3%. EBV positivity was detected in 50.7% of patients.

As shown in **Table 2**, clinical characteristics differed significantly across IEI subgroups. Sex distribution varied among groups (p = 0.001), with a marked male predominance in the ID subgroup (100%). Recurrent infections were significantly more frequent in patients with DNA repair defects (81.8%) and CID (63.0%) compared with PAD (23.1%) and ID (33.3%) (p = 0.009). Parental consanguinity also differed across subgroups (p = 0.025), being most prevalent in DNA repair defects (100%) and CID (74.1%). EBV positivity (tissue and/or serum) showed a significant association with IEI category (p = 0.011), with the highest rate observed in the ID subgroup (86.7%). In contrast, no statistically significant differences were observed between IEI subgroups regarding bronchiectasis, family history, autoimmunity, or lymphoproliferative manifestations (all p > 0.05).

Immunological evaluation revealed lymphopenia in 47.5% of patients. Hypogammaglobulinemia was present in 70.5%, with IgG deficiency in 51.7%, IgM deficiency in 50.8%, and IgA deficiency in 41.0%. Isohemagglutinin responses were absent in 30.8%, and impaired vaccine responses were documented in 28.8%. Reduced CD3+, CD4+, and/or CD19+, CD20+ counts were observed in accordance with the underlying IEI subtypes.

Exploratory comparison of lymphocyte subsets included 24 HL and 36 NHL cases; sample sizes varied by marker because of missing data. Naïve Th percentages were higher in NHL than in HL [51.0% (IQR, 29.7–59.0) vs. 20.0% (9.7–32.3); unadjusted p = 0.013], but this difference did not remain statistically significant after Benjamini–Hochberg correction for multiple testing (adjusted p = 0.076). Absolute naïve T-cell counts were numerically higher in NHL than in HL [315.9 (99.8–563.0) vs. 142.1 (47.8–368.5); unadjusted p = 0.063, adjusted p = 0.164]. Naïve Tc percentages were also numerically higher in NHL but were not significant [45.0% (34.5–57.0) vs. 28.0% (22.4–41.5); unadjusted p = 0.082, adjusted p = 0.164]. CD3, CD4, and CD19/20 levels did not differ significantly between HL and NHL. Overall, none of the evaluated immunophenotypic markers was significantly associated with lymphoma type after multiple-testing correction.

Among patients with available EBV results, tissue EBV positivity was detected in 25 of 53 patients (47.2%), while serum EBV positivity was detected in 27 of 54 patients (50.0%). Tissue EBV-positive and EBV-negative patients had similar HL/NHL distributions (p = 1.000), frequencies of advanced-stage disease (88.0% vs. 81.8%, p = 0.690), and ages at malignancy diagnosis [median 9.0 vs. 11.5 years, p = 0.437]. Rituximab use was numerically higher among tissue EBV-positive patients (68.0% vs. 48.1%), although the difference was not significant (p = 0.171). Similarly, serum EBV positivity was not associated with HL/NHL distribution (p = 0.770), advanced-stage disease (92.6% vs. 81.0%, p = 0.383), or rituximab use (66.7% vs. 50.0%, p = 0.271). Serum EBV-positive patients were numerically younger at malignancy diagnosis [median 9.0 vs. 12.0 years], but this difference was not significant (p = 0.093). Neither tissue nor serum EBV positivity was associated with HSCT, mortality, or overall survival (log-rank p = 0.647 and p = 0.550, respectively). None of the comparisons remained significant after correction for multiple testing. Among patients with EBV positivity in tissue and/or serum, mortality was similar between those who received rituximab and those who did not (33.3% [7/21] vs. 36.4% [4/11], Fisher’s exact p = 1.000).

### Treatment

Treatment was individualized according to tumor histology and underlying IEI. Solid tumors were managed primarily with surgery with or without chemotherapy, whereas hematologic malignancies were treated according to disease-specific protocols. Detailed treatment data are provided in **Supplementary Table 1**.

Radiotherapy was administered to seven patients with bulky disease, all diagnosed with HL.

Rituximab was administered to 34 patients (50.7%) at least once during the disease course, including first-line use in 27 patients (40.3%) and second-line or salvage use in 13 patients (19.4%). Rituximab was administered to the majority of patients with CD20-positive B-cell lymphomas (22/25, 88%). Brentuximab vedotin was used in seven patients (10.6%), exclusively in the salvage setting, and was administered to patients with CD30-positive lymphomas. No severe treatment-related toxicities attributable to rituximab or brentuximab vedotin were documented in the available medical records.

Among the 11 patients with DNA repair defects, 10 had ataxia-telangiectasia and one had Nijmegen breakage syndrome. All received modified chemotherapy with dose reduction or reduced treatment intensity compared with standard protocols, reflecting the anticipated vulnerability of this subgroup to genotoxic therapy. Radiotherapy was avoided in all patients with DNA repair defects. Treatment regimens were heterogeneous and varied according to malignancy subtype, treatment era, and clinical condition, including ABVD-, R-CHOP-, BFM/POG-based, and relapse-directed approaches. Despite treatment adaptation, 8 of 11 patients died during follow-up. One patient developed severe mucositis during treatment and died from sepsis.

Twelve patients underwent allogeneic HSCT, seven in first remission and five in second remission. Among the 11 patients with recorded numeric follow-up, the median follow-up after HSCT was 80 months (IQR, 57; range, 3–151); one additional patient died within the first month. Chronic GVHD developed in four patients, and no post-transplant relapses were observed (**Table 3**).

**Table 3:** Characteristics and outcomes of patients with IEI who underwent allogeneic hematopoietic stem cell transplantation.

Patient no.	IEI diagnosis	Donor type	Conditioning regimen	GVHD prophylaxis	Chronic GVHD	T-cell chimerism (%)*	Follow-up after HSCT (months)	Relapse after HSCT
1	CD70 deficiency	MSD	Treosulfan/fludarabine/rituximab	CsA/MMF	Skin/GI	100	84	No
2	GIMAP5	MSD	Treosulfan/fludarabine/thiotepa/rituximab	CsA/MTX	None		Died within first month	No
3	DCLRE1C	MUD	Treosulfan/fludarabine	ATG/CsA/MMF	GI	100	75	No
4	RASGRP1	MSD	Treosulfan/fludarabine	Tacrolimus/MMF (IV)	None	99	68	No
5	CD70 deficiency	MUD	Treosulfan/fludarabine/rituximab	ATG/CsA/MMF	Pulmonary	99	84	No
6	CD70 deficiency	MUD	Treosulfan/fludarabine/rituximab	ATG/CsA/MMF	None	100	80	No
7	DCLRE1C	MSD	Treosulfan/fludarabine/rituximab	Tacrolimus/MMF	Skin/GI	98	52	No
8	ITK	MUD	Treosulfan/fludarabine/rituximab	ATG/CsA/MMF	None	95	109	No
9	CID	MSD	Treosulfan/fludarabine/rituximab	Tacrolimus/MTX	None	99	3	No
10	IL-2R deficiency	Haploidentical (father)	None	PTCy/tacrolimus/MTX	None	95	21	No
11	DOCK8	MSD	Treosulfan/fludarabine	CsA	None	100	151	No
12	DOCK8	9/10 MMUD	Treosulfan/fludarabine	ATG/CsA/MMF	None	98	121	No

* T-cell chimerism is expressed as the percentage of donor-derived T cells at last follow-up.Abbreviations: ATG, anti-thymocyte globulin; CsA, cyclosporine A; GI, gastrointestinal; GVHD, graft-versus-host disease; HSCT, hematopoietic stem cell transplantation; IEI, inborn errors of immunity; MMF, mycophenolate mofetil; MMUD, mismatched unrelated donor; MSD, matched sibling donor; MTX, methotrexate; MUD, matched unrelated donor; PTCy, post-transplant cyclophosphamide.

### Mortality and outcome

At last follow-up, 41 patients (61.2%) were alive and 26 (38.8%) had died. Disease-related events (relapse and/or progression) occurred in 32 patients (47.8%). Median EFS was 66 months (95% CI, 11.0-121.0) and median OS was 155 months (95% CI, 102.8-207.2). Among deaths, 23 of 26 (88.5%) were attributable to malignancy.

### Survival analysis

**Figure 3:**
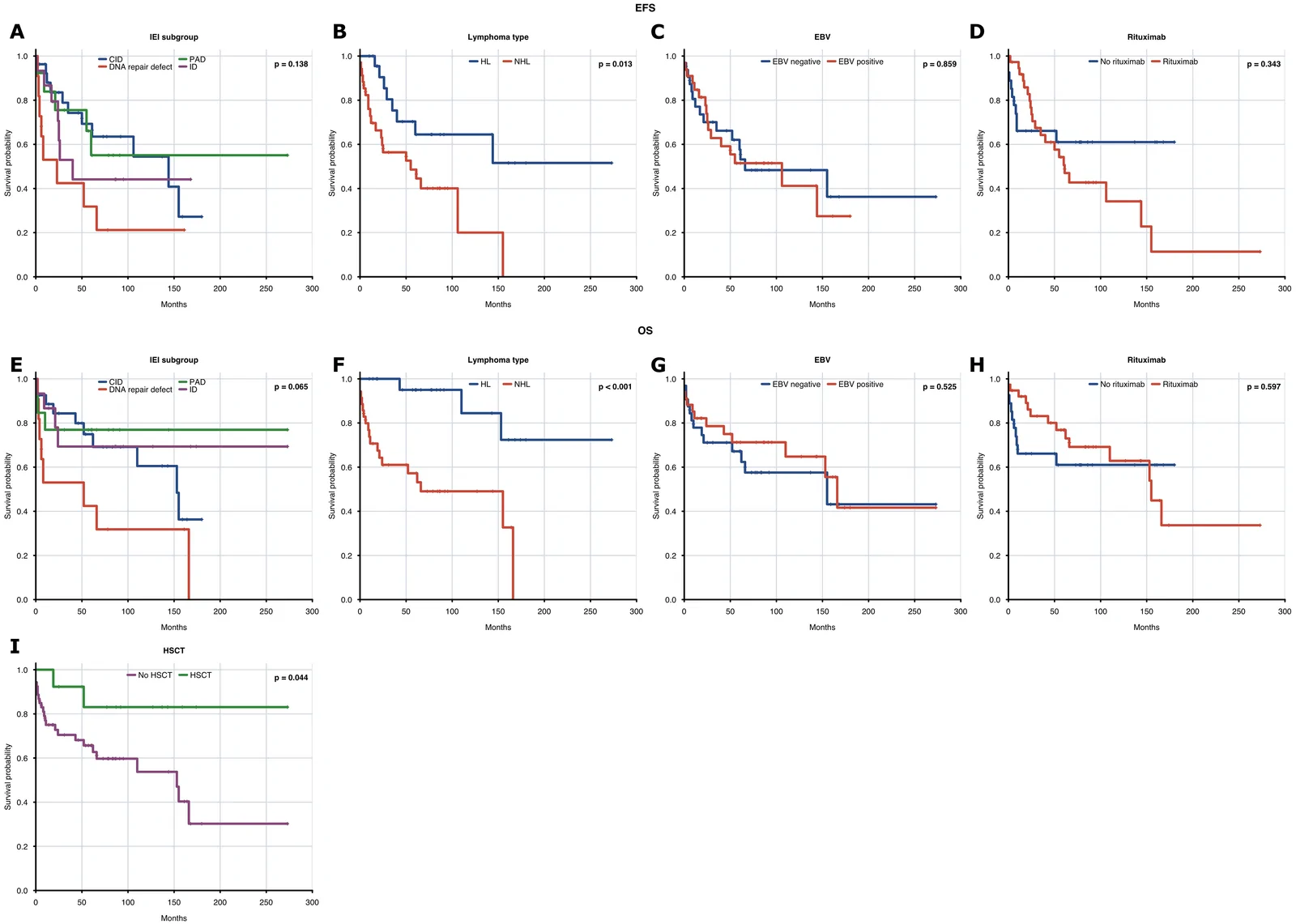
Kaplan-Meier event-free survival and overall survival curves in patients with inborn errors of immunity and malignancy. Panels **(A-D)** show event-free survival. **(A)** Event-free survival according to inborn error of immunity subgroup, showing no statistically significant difference among groups, although the DNA repair defect subgroup had the poorest event-free survival and the predominantly antibody deficiency subgroup showed the most favorable trend (p = 0.138). **(B)** Event-free survival according to lymphoma type, demonstrating significantly better event-free survival in Hodgkin lymphoma than in non-Hodgkin lymphoma (p = 0.013). **(C)** Event-free survival according to Epstein-Barr virus status, showing no significant difference between virus-positive and virus-negative patients (p = 0.859). **(D)** Event-free survival according to rituximab use, showing no statistically significant difference between patients who received rituximab and those who did not (p = 0.343). Panels **(E-I)** show overall survival. **(E)** Overall survival according to inborn error of immunity subgroup showed a borderline difference (log-rank p = 0.065), with the poorest survival observed in patients with DNA repair defects, whereas predominantly antibody deficiency and immune dysregulation showed relatively more favorable survival; the combined immunodeficiency subgroup showed a more pronounced decline during later follow-up. **(F)** Overall survival was significantly better in patients with Hodgkin lymphoma than in those with non-Hodgkin lymphoma (p < 0.001). **(G)** Overall survival did not differ significantly according to Epstein-Barr virus status (p = 0.525). **(H)** Rituximab use was not associated with a significant difference in overall survival (p = 0.597). **(I)** Overall survival appeared higher among patients who underwent hematopoietic stem cell transplantation than among those who did not (p = 0.044), but this unadjusted comparison should be interpreted cautiously because transplant recipients were few and clinically selected.

Kaplan-Meier analyses showed no significant EFS or OS difference across IEI subgroups, although patients with DNA repair defects tended to have poorer OS. By malignancy type, patients with HL had significantly better outcomes than those with NHL, with superior EFS (p = 0.013; 5-year EFS approximately 63% vs 38%) and OS (p < 0.001; 5-year OS approximately 95% vs 49%). EBV status and rituximab use were not associated with outcome. Although HSCT was not associated with better EFS, no relapse occurred after transplantation, and OS appeared higher in transplanted patients than in non-transplanted patients in unadjusted Kaplan-Meier analysis (p = 0.044) (**Figure 3**). Because only 12 patients underwent HSCT and transplantation was performed in selected patients who had achieved remission, this association was considered exploratory and potentially affected by selection bias.

Among surviving patients, the median follow-up duration from malignancy diagnosis to last contact was 79 months (IQR, 50-137).

### Univariable analysis

In univariable Cox analysis, malignancy type was the variable most strongly associated with OS (global p < 0.001 in the three-category model). Compared with HL, both NHL (HR, 7.16; 95% CI, 2.06-24.81; p = 0.002) and solid/plasma cell tumors (HR, 7.80; 95% CI, 1.70-35.85; p = 0.008) were associated with higher mortality. DNA repair defect status was also associated with worse OS in univariable analysis (HR, 2.72; 95% CI, 1.18-6.29; p = 0.019). Age at IEI diagnosis (HR per year, 1.00; 95% CI, 0.94-1.06; p = 0.971) and age at malignancy diagnosis (HR per year, 1.04; 95% CI, 0.98-1.10; p = 0.210) were not significantly associated with OS (**Supplementary Table 2**).

To explore potential era effects, patients were stratified according to the calendar period of malignancy diagnosis as early era (<=2012) and late era (>=2013). Among 61 patients with available malignancy diagnosis dates, 16 were classified in the early era and 45 in the late era. Overall survival did not differ significantly between eras (log-rank p = 0.473; HR for late vs. early era, 1.46; 95% CI, 0.52-4.09; p = 0.473).

### Multivariable analysis

Age at IEI diagnosis and age at malignancy diagnosis were strongly correlated (Pearson r = 0.83; Spearman’s rho = 0.67, p < 0.001) and were therefore not entered simultaneously. Age at malignancy diagnosis was retained in the multivariable model because OS follow-up began at malignancy diagnosis. In the clinically selected multivariable Cox model including malignancy type, age at malignancy diagnosis, and DNA repair defect status, all three factors were associated with OS. Compared with HL, NHL was associated with higher mortality (HR, 7.43; 95% CI, 2.15-25.71; p = 0.002), as were solid/plasma cell tumors (HR, 6.12; 95% CI, 1.29-29.06; p = 0.023). Older age at malignancy diagnosis was associated with worse OS (HR per year, 1.07; 95% CI, 1.01-1.13; p = 0.016), and DNA repair defect status remained associated with worse OS compared with other IEI groups (HR, 2.73; 95% CI, 1.10-6.80; p = 0.031) (**Supplementary Table 2**). The proportional hazards assumption was not violated (global Schoenfeld test, p = 0.30).

## Discussion

In this single-center cohort of 67 patients with IEI complicated by malignancy, we provide a detailed clinicopathological and outcome-focused analysis in a predominantly pediatric and adolescent population. Our results show that malignancy in IEI is predominantly lymphoid, frequently precedes the diagnosis of the underlying immune disorder, and often presents at an advanced stage. We further demonstrate marked heterogeneity in cancer burden across IEI subgroups, with the highest proportions observed in PAD, DNA repair defects, and ID. In the clinically selected primary multivariable model, NHL, solid/plasma cell tumors, older age at malignancy diagnosis, and DNA repair defect status were associated with worse OS.

The proportion of IEI patients developing malignancy in our cohort (4%) is broadly consistent with registry-based data, although direct comparison is limited by differences in cohort composition and follow-up duration. The ESID registry analysis of nearly 20,000 patients confirmed that malignancies occur across all major IEI categories, with an overall malignancy rate of 9%; PAD contributed the largest absolute number of affected patients, while syndromic disorders and immune dysregulation-related diseases also carried a substantial cancer burden (10). The lower proportion observed in our study may partly reflect the predominantly pediatric nature of our cohort. Similarly, the USIDNET registry demonstrated a significantly increased overall cancer risk in patients with IEI, while common solid tumors are not overrepresented (3). In line with these observations, lymphoid malignancies accounted for 90% of all cancers in our cohort, consistent with the central role of defective immune surveillance and intrinsic defects in oncogenesis.

A molecular/genetic diagnosis was established in 67.2% of our cohort, a rate toward the upper end of the variable diagnostic yields reported in IEI studies. Previous studies have reported a WES-based diagnostic rate of 41.1% in a large multicenter cohort from Türkiye and a diagnostic rate of 56% in 878 patients evaluated using targeted-panel and WES-based strategies (15, 16) In a cohort consisting predominantly of patients with sporadic antibody deficiency, the targeted-panel yield was 15.3% overall and increased to 48.6% among patients with consanguinity or a family history of IEI (17). The comparatively high molecular diagnosis rate in our cohort may reflect its clinically selected nature, the presence of consanguinity and familial cases, the predominance of established IEI phenotypes complicated by malignancy, and the use of single-gene testing in patients with a strong preliminary diagnosis. Therefore, the overall rate should not be interpreted as the diagnostic performance of a single platform, because testing methods and selection criteria varied over the 21-year study period. Nevertheless, the 32.8% of patients who remained without a molecular diagnosis highlights the potential value of future reanalysis using updated genomic approaches.

In our cohort, PAD showed the highest subgroup-specific malignancy proportion, with malignancy observed in 13 of 49 PAD patients (26.5%). This finding should be interpreted cautiously and should not be considered a population-based incidence estimate. Our center is a tertiary referral center where IEIs potentially correctable by HSCT, including CID, ID, and DNA repair defects, are frequently referred; therefore, the relative number of PAD patients in the overall IEI cohort may be lower than expected, which could inflate the apparent malignancy proportion within this subgroup. Nevertheless, this observation is broadly consistent with large registry data showing that predominantly antibody deficiencies, particularly CVID/CVID-like disorders, contribute substantially to the overall malignancy burden in IEI (3, 10). In PAD, cancer susceptibility is likely multifactorial and may reflect abnormal B-cell maturation, chronic lymphoproliferation, immune dysregulation, and impaired control of oncogenic infections (18–20).

In our PAD subgroup, the combination of universal hypogammaglobulinemia, less frequently documented recurrent infections, and a high rate of malignancy-first presentation suggests that clinically less conspicuous antibody deficiency may remain unrecognized until malignancy prompts immunologic evaluation. Thus, this pattern may reflect a combination of intrinsic immune dysregulation, delayed recognition of antibody deficiency, and referral/ascertainment bias rather than a single PAD-specific tumor pathway. Although Burkitt lymphoma was numerically more frequent among PAD patients, this exploratory finding did not remain significant after correction and should be considered hypothesis-generating.

The less favorable outcomes observed in patients with DNA repair defects are clinically meaningful and biologically plausible. These disorders confer intrinsic cancer susceptibility through impaired genomic stability and may also increase vulnerability to treatment-related toxicity (21–23). In our cohort, all patients with DNA repair defects received dose-reduced or reduced-intensity chemotherapy, radiotherapy was avoided, and 8 of 11 patients died during follow-up. Therefore, outcome in this subgroup may have been shaped by aggressive tumor biology, limited tolerance to standard-intensity oncologic therapy, treatment de-intensification, infectious complications, or a combination of these factors. However, because detailed delivered dose intensity, cumulative chemotherapy exposure, and toxicity-driven modifications were not systematically captured, the independent effect of treatment modification on survival could not be determined.

A particularly important finding of our study is that malignancy preceded the diagnosis of IEI in approximately two-thirds of patients. This is consistent with registry-based and pediatric single-center reports showing that cancer may be the first manifestation of an underlying immune defect (10, 24, 25). We further demonstrate a clear age dependency: malignancy-first presentation was especially common in adolescents, whereas patients diagnosed at younger ages were more likely to have IEI recognized before cancer. Importantly, patients in whom malignancy preceded IEI diagnosis were more likely to present with advanced-stage disease. These findings underscore the need for close oncological–immunological collaboration in children and adolescents with lymphoma. At our center, this collaboration often enabled assessment before chemotherapy, improving characterization of the underlying immune phenotype (22, 25). Therefore, the high rate of malignancy-first diagnosis in our cohort should not be interpreted solely as diagnostic delay but also as a reflection of proactive case identification enabled by integrated oncological–immunological care.

Accordingly, oncologists should consider immunological evaluation in patients with recurrent, severe, or unusual infections; bronchiectasis; autoimmunity; persistent lymphoproliferation or EBV viremia; consanguinity; or a family history of IEI, malignancy, severe infections, or unexplained childhood deaths (18, 26). Early-onset, unusual, extranodal, multifocal, virus-associated, recurrent, or treatment-refractory malignancy may provide additional clues (21). Disproportionate chemotherapy toxicity, prolonged cytopenia, severe infectious complications, or unexpected radiosensitivity should likewise prompt referral to a clinical immunologist (22, 27). Importantly, whenever clinically feasible, immunological evaluation should be completed before chemotherapy is initiated, because treatment-related changes in immunoglobulin concentrations and lymphocyte subsets may obscure the baseline immune phenotype and complicate recognition of an underlying IEI. Initial evaluation may include a complete blood count with differential, quantitative immunoglobulins, lymphocyte subsets, and targeted genetic testing when indicated (9, 21).

The lymphoma spectrum in our cohort, dominated by aggressive NHL and classical HL, closely resembled that reported in registry-based and single-center series (6, 20, 28, 29). Extranodal disease predominated in NHL, whereas HL more frequently presented with nodal involvement, reflecting known biological differences. Recent genomic investigations have shown that lymphomas associated with IEI are not simply sporadic cases occurring in immunocompromised individuals, but rather constitute biologically unique entities (27). These molecular insights provide a biological framework for the aggressive clinical behavior and atypical presentations frequently observed in this patient population.

Although EBV is an established oncogenic cofactor in IEI-associated lymphomas, its prognostic significance may vary according to age, lymphoma subtype, underlying immune defect, and the method used for EBV detection (18–20, 30). In our predominantly pediatric and adolescent cohort, neither tissue nor serum EBV positivity was associated with lymphoma subtype, disease stage, treatment exposure, mortality, or overall survival. This lack of association may reflect the heterogeneity of underlying IEIs and lymphoma histologies, limited subgroup sizes, missing EBV data, and the greater influence of immune dysfunction, infectious complications, and treatment-related toxicity on outcome in immunodeficient patients. Numerically greater rituximab use among EBV-positive patients may also have attenuated a potential adverse effect; however, the small, nonrandomized treatment subgroups preclude any causal inference regarding the effect of rituximab on mortality. Therefore, our findings do not exclude a role for EBV in lymphomagenesis but suggest that EBV positivity alone was not a prognostic marker in this cohort.

Our survival analyses identified malignancy type as the strongest tumor-related prognostic factor, with worse outcomes among patients with NHL and solid/plasma cell tumors than among those with HL (10). In the primary multivariable model, older age at malignancy diagnosis and DNA repair defect status were also associated with worse OS, supporting the relevance of both oncological context and underlying IEI-related vulnerability (21, 23). In particular, patients with DNA repair defects had a higher adjusted hazard of death than patients with other IEI categories (HR, 2.73; 95% CI, 1.10-6.80), although this estimate should be interpreted cautiously given the small subgroup size. Because age at IEI diagnosis and age at malignancy diagnosis represent related aspects of diagnostic timing, age at IEI diagnosis was not included in the multivariable model, thereby avoiding unstable estimates from collinearity. Despite frequently presenting at advanced stages, HL was associated with more favorable outcomes. These findings suggest that classical oncological factors, particularly tumor type and age at cancer diagnosis, remain central to outcome in IEI-associated malignancy, while host genetic context may further modify risk.

Although lymphoma treatment evolved substantially during the 21-year study period, OS did not differ significantly between the early and late diagnostic eras in our cohort. Although exploratory, this finding is clinically informative and may suggest that advances in lymphoma therapy do not fully overcome the host-related vulnerabilities inherent to IEI-associated malignancy. It should therefore be interpreted cautiously and not generalized as evidence that contemporary lymphoma protocols or rituximab lack benefit. In the general pediatric population, rituximab has improved outcomes in high-risk mature B-cell NHL; however, outcomes in IEI-associated malignancy are also shaped by host-related factors, including the underlying immune defect, infection susceptibility, immune dysregulation, organ damage, and treatment-related toxicity (31). Recent ESID registry data show that oncologic treatment is frequently modified because of IEI/PID and that IEI/PID is perceived to adversely affect complications and outcomes (10). Similarly, children with NHL and pre-existing conditions, including DNA repair defects and primary immunodeficiencies, have inferior survival and substantial treatment-related mortality compared with children without such conditions (10, 32). Patients with DNA damage response defects may be particularly vulnerable to genotoxic therapy. Therefore, the absence of an era-related survival difference in our cohort is best viewed as a hypothesis-generating observation that highlights the need to consider persistent host-related vulnerability, advanced-stage presentation, heterogeneous IEI and malignancy subtypes, and non-randomized treatment allocation when interpreting temporal trends in this population (21).

Allogeneic HSCT offers definitive management for selected IEI patients by correcting the underlying immune defect while consolidating oncologic control (21, 28). In our cohort, only 12 patients underwent HSCT, most after achieving remission. Although OS appeared more favorable among transplanted patients and no post-transplant relapse was observed, this finding should be interpreted with substantial caution. Transplant recipients represent a selected subgroup with sufficient disease control, clinical stability, donor availability, and survival time to proceed to HSCT; therefore, transplant eligibility, indication bias, and immortal time bias may all contribute to the observed OS difference. Accordingly, the unadjusted survival association should not be interpreted as causal evidence that HSCT independently improves OS in this cohort. Nevertheless, the absence of post-transplant relapse suggests that, in carefully selected patients, HSCT may contribute to durable disease control.

Limitations of our study include its retrospective, single-center design, the relatively small numbers within specific IEI and tumor subgroups, and the long study period encompassing changes in diagnostic and therapeutic strategies. The high rate of parental consanguinity may limit generalizability to outbred populations, and the relatively high malignancy proportions observed in some IEI subgroups, particularly PAD, should be interpreted cautiously because referral and ascertainment bias are possible in a tertiary center. Treatment-related analyses were limited by retrospective documentation; although all patients with DNA repair defects received dose-reduced or reduced-intensity chemotherapy and radiotherapy was avoided, exact dose intensity, cumulative chemotherapy exposure, dose delays, and toxicity-driven modifications were not captured in a standardized manner. Similarly, the absence of documented severe toxicity attributable to rituximab or brentuximab vedotin should not be interpreted as definitive evidence that no toxicity occurred. Exploratory lymphocyte subset and EBV-related analyses were limited by missing data, small subgroup sizes, lack of standardized quantitative or longitudinal EBV viral-load measurements, and the inability to adjust rituximab-related mortality comparisons for treatment indication and other prognostic confounders. Genetic testing was performed over a long study period using different platforms and diagnostic centers, which may have limited the comparability of diagnostic approaches and molecular diagnostic yield across patients. Finally, the era-based survival analysis was limited by the small number of early-era patients. Nevertheless, the detailed phenotypic, immunological, and oncological characterization of this cohort, together with long-term follow-up and comprehensive survival analyses, represents an important strength and allows meaningful comparison with registry-based and mechanistic studies.

In conclusion, malignancy in patients with IEI was predominantly lymphoid and frequently represented the first recognized manifestation of the underlying immune disorder. Outcomes were associated with malignancy type, age at malignancy diagnosis, and DNA repair defect status, highlighting the combined influence of tumor-related and host-related factors. These findings support early immunologic evaluation—whenever feasible before chemotherapy—and multidisciplinary, individualized management of children and adolescents presenting with malignancy, particularly lymphoma. HSCT may provide durable disease control in carefully selected patients, although its apparent survival association in this retrospective cohort should be interpreted cautiously.

## Data Availability

The datasets presented in this article are not readily available because they contain potentially identifiable clinical information and are subject to ethical and institutional restrictions. Requests to access the datasets should be directed to the corresponding author, Zehra Sule Haskologlu, at haskologlu@ankara.edu.tr.

## References

[1] Poli MC Aksentijevich I Bousfiha AA Cunningham-Rundles C Hambleton S Klein C . Human inborn errors of immunity: 2024 update on the classification from the International Union of Immunological Societies Expert Committee. J Hum Immun. (2025) 1:e20250003. doi: 10.70962/jhi.20250003 41608114 PMC12829761

[2] Seleman M Hoyos-Bachiloglu R Geha RS Chou J . Uses of next-generation sequencing technologies for the diagnosis of primary immunodeficiencies. Front Immunol. (2017) 8:847. doi: 10.3389/fimmu.2017.00847 28791010 PMC5522848

[3] Mayor PC Eng KH Singel KL Abrams SI Odunsi K Moysich KB . Cancer in primary immunodeficiency diseases: cancer incidence in the USIDNET Registry. J Allergy Clin Immunol. (2018) 141:1028–35. doi: 10.1016/j.jaci.2017.05.024 28606585 PMC5723251

[4] Filipovich AH Mathur A Kamat D Shapiro RS . Primary immunodeficiencies: genetic risk factors for lymphoma. Cancer Res. (1992) 52:5465s–7s. 1327508

[5] Salavoura K Kolialexi A Tsangaris G Mavrou A . Development of cancer in patients with primary immunodeficiencies. Anticancer Res. (2008) 28:1263–9. 18505064

[6] Leechawengwongs E Shearer WT . Lymphoma complicating primary immunodeficiency syndromes. Curr Opin Hematol. (2012) 19:305–12. doi: 10.1097/MOH.0b013e328353fa13 22525579

[7] Mueller BU Pizzo PA . Cancer in children with primary or secondary immunodeficiencies. J Pediatr. (1996) 126:1–10. doi: 10.1016/s0022-3476(95)70491-4 7815195

[8] Suarez F Lortholary O Hermine O Lecuit M . Infection-associated lymphomas derived from marginal zone B cells: a model of antigen-driven lymphoproliferation. Blood. (2006) 107:3034–44. doi: 10.1182/blood-2005-09-3679 16397126

[9] Shapiro RS . Malignancies in the setting of primary immunodeficiency: implications for hematologists/oncologists. Am J Hematol. (2011) 86:48–55. doi: 10.1002/ajh.21903 21120868

[10] Bogaert DJA Wolfsberger CH Attarbaschi A Gathmann J Warnatz K Mueller G . Epidemiology and management of Malignancies in patients with inborn errors of immunity: an ESID registry study of 19,959 patients. J Allergy Clin Immunol. (2026) 157:739–53. doi: 10.1016/j.jaci.2025.10.033 41242640

[11] Seidel MG Kindle G Gathmann B Quinti I Buckland M van Montfrans J . The European Society for Immunodeficiencies Registry working definitions for the clinical diagnosis of inborn errors of immunity. J Allergy Clin Immunol Pract. (2019) 7:1763–70. doi: 10.1016/j.jaip.2019.02.004 30776527

[12] Alaggio R Amador C Anagnostopoulos I Attygalle AD Araujo IBO Berti E . The 5th edition of the World Health Organization Classification of Haematolymphoid Tumours: lymphoid neoplasms. Leukemia. (2022) 36:1720–48. doi: 10.1038/s41375-022-01620-2 35732829 PMC9214472

[13] Campo E Jaffe ES Cook JR Quintanilla-Martinez L Swerdlow SH Anderson KC . The international consensus classification of mature lymphoid neoplasms: a report from the clinical advisory committee. Blood. (2022) 140:1229–53. doi: 10.1182/blood.2022015851 35653592 PMC9479027

[14] Halacli SO Ayvaz DC Sun-Tan C Erman B Uz E Yilmaz DY . STK4 (MST1) deficiency in two siblings with autoimmune cytopenias: a novel mutation. Clin Immunol. (2015) 161:316–23. doi: 10.1016/j.clim.2015.06.010 26117625

[15] Platt CD Zaman F Bainter W Stafstrom K Almutairi A Reigle M . Efficacy and economics of targeted panel versus whole-exome sequencing in 878 patients with suspected primary immunodeficiency. J Allergy Clin Immunol. (2021) 147:723–6. doi: 10.1016/j.jaci.2020.08.022 32888943 PMC7870529

[16] Erman B Aba U Ipsir C Pehlivan D Aytekin C Cildir G . Genetic evaluation of patients with clinically diagnosed inborn errors of immunity by whole-exome sequencing: results from a specialized research center for immunodeficiency in Türkiye. J ClinImmunol. (2024) 44:157. doi: 10.1007/s10875-024-01759-w 38954121 PMC11219406

[17] Sogkas G Dubrowinskaja N Schütz K Steinbrück L Götting J Schwerk N . Diagnostic yield and therapeutic consequences of targeted next-generation sequencing in sporadic primary immunodeficiency. Int Arch Allergy Immunol. (2022) 183:337–49. doi: 10.1159/000519199 34619682 PMC8985027

[18] Shabani M Nichols KE Rezaei N . Primary immunodeficiencies associated with EBV-induced lymphoproliferative disorders. Crit Rev Oncol Hematol. (2016) 108:109–27. doi: 10.1016/j.critrevonc.2016.10.014 27931829

[19] Eminger LA Hall LD Hesterman KS Heymann WR . Epstein–Barr virus: dermatologic associations and implications: part II. Associated lymphoproliferative disorders and solid tumors. J Am Acad Dermatol. (2015) 72:21–34. doi: 10.1016/j.jaad.2014.07.035 25497918

[20] Duan L Grunebaum E . Hematological Malignancies associated with primary immunodeficiency disorders. Clin Immunol. (2018) 194:46–59. doi: 10.1016/j.clim.2018.06.011 29966714

[21] Bomken S van der Werff Ten Bosch J Attarbaschi A Bacon CM Borkhardt A Boztug K . Current understanding and future research priorities in Malignancy associated with inborn errors of immunity and DNA repair disorders. Front Immunol. (2018) 9:2912. doi: 10.3389/fimmu.2018.02912 30619276 PMC6299915

[22] Fournier B Mahlaoui N Moshous D de Villartay JP . Inborn errors of immunity caused by defects in the DNA damage response pathways: importance of minimizing treatment-related genotoxicity. Pediatr Allergy Immunol. (2022) 33:e13820. doi: 10.1111/pai.13820 35754136 PMC9327728

[23] Taylor AM Metcalfe JA Thick J Mak YF . Leukemia and lymphoma in ataxia telangiectasia. Blood. (1996) 87:423–38. doi: 10.1182/blood.V87.2.423.bloodjournal872423 8555463

[24] Thalhammer J Kindle G Nieters A Rusch S Seppänen MRJ Fischer A . Initial presenting manifestations in 16,486 patients with inborn errors of immunity. J Allergy Clin Immunol. (2021) 148:1332–1341.e5. doi: 10.1016/j.jaci.2021.04.015 33895260

[25] Maffeis M Notarangelo LD Schumacher RF Soncini E Soresina A Lanfranchi A . Primary immunodeficiencies and oncological risk: the experience of the Children’s Hospital of Brescia. Front Pediatr. (2019) 7:232. doi: 10.3389/fped.2019.00232 31275905 PMC6593615

[26] Hauck F Voss R Urban C Seidel MG . Intrinsic and extrinsic causes of Malignancies in patients with primary immunodeficiency disorders. J Allergy Clin Immunol. (2018) 141:59–68.e4. doi: 10.1016/j.jaci.2017.06.009 28669558

[27] Ye X Maglione PJ Wehr C Li X Wang Y Abolhassani H . Genomic characterization of lymphomas in patients with inborn errors of immunity. Blood Adv. (2022) 6:5403–14. doi: 10.1182/bloodadvances.2021006654 35687490 PMC9631701

[28] Genc Ozbay Z Soyak Aytekin E Esenboga S Cagdas D . Malignancies in the context of inborn errors of immunity: an immunologist’s view. Expert Rev Clin Immunol. (2026) 22:223–31. doi: 10.1080/1744666X.2026.2633137 41693687

[29] Cekic S Metin A Aytekin C Edeer Karaca N Baris S Karali Y . The evaluation of Malignancies in Turkish primary immunodeficiency patients: a multicenter study. Pediatr Allergy Immunol. (2020) 31:528–36. doi: 10.1111/pai.13231 32060950

[30] Hu J Zhang X Tao H Jia Y . The prognostic value of Epstein–Barr virus infection in Hodgkin lymphoma: a systematic review and meta-analysis. Front Oncol. (2022) 12:1034398. doi: 10.3389/fonc.2022.1034398 36387159 PMC9648611

[31] Minard-Colin V Aupérin A Pillon M Burke GAA Barkauskas DA Wheatley K . Rituximab for high-risk, mature B-cell non-Hodgkin lymphoma in children. N Engl J Med. (2020) 382:2207–19. doi: 10.1056/NEJMoa1915315 32492302 PMC7720281

[32] Attarbaschi A Carraro E Abla O Barzilai-Birenboim S Bomken A Brugières L . Non-Hodgkin lymphoma and pre-existing conditions: spectrum, clinical characteristics and outcome in 213 children and adolescents. Haematologica. (2016) 101:1581–91. doi: 10.3324/haematol.2016.147116 27515251 PMC5479624

