# Supplementary material for: Clinical spectrum and survival outcomes of malignancies in pediatric patients with inborn errors of immunity
Source: Front Immunol. 2026 Aug 14;17:1904173. doi: 10.3389/fimmu.2026.1904173 (PMC13521861; doi:10.3389/fimmu.2026.1904173)

Table S1. **Clinical and Outcome Summary of Patients With Inborn Errors of Immunity and Malignancy**

Patient	Sex	Age at diagnosis (IEI / malignancy)	IEI type / IEI category	Histopathologic diagnosis of malignancy	Stage	Biopsy Site	EBV status (serum PCR/tissue EBER)	Treatment modality / regimen	Immunotherapy	Status
1	M	13 / 13	CD70 deficiency / ID	NHL / Aggressive B-cell lymphoma	3B	Cervical	+/+	CHOP	RTX; BV	Relapse
2	M	4 / 4	LRBA / ID	NHL / T-cell lymphoma	3A	Axillary	+/+	Vincristine + cyclophosphamide + prednisolone	None	PD
3	M	4 / 3	Ataxia-telangiectasia / DNA repair defect	NHL / Aggressive B-cell lymphoma	4A	Intracranial	+/+	CHOP	RTX	PD
4	M	11 / 13	GIMAP5 / CID	HL / mixed cellularity subtype*	4A	Abdomen	+/+	ABVD → Bendamustine	RTX; BV	Relapse, PD
5	F	15 / 10	CVID / PAD	NHL / Burkitt lymphoma	4A	Abdomen	+/+	POG 9317	RTX; BV	Relapse
6	F	4 / 13	DCLRE1C / CID	NHL / Marginal zone lymphoma	2E	Supraclavicular	-/-	None	RTX	CR

7	M	14 / 14	CTLA4 deficiency / ID	NHL / Burkitt lymphoma	4A	Abdomen	+/+	POG 9317	RTX	CR
8	M	8 / 8	RASGRP1 / ID	HL / mixed cellularity subtype	3A	Cervical	+/+	ABVD	RTX	CR
9	M	14 / 14	CVID / PAD	HL / mixed cellularity subtype	2A	Cervical	+/-	ABVD	RTX	CR
10	M	14 / 3	CD70 deficiency / ID	HL / mixed cellularity subtype	4A	Salivary gland	-/+	OPPA → COPP → ABVD	RTX	Relapse, PD
11	M	8 / 3	CD70 deficiency / ID	HL / mixed cellularity subtype	4A	Cervical	+/-	ABVD	None	Relapse, PD
12	M	12 / 6	CD70 deficiency / ID	NHL / Burkitt lymphoma	3A	NA	+/+	NHL-BFM95 AA/BB/CC	None	CR
13	M	17 / 17	GIMAP5 / CID	HL / nodular sclerosis subtype	4B	Colon	+/+	ABVD → GDP	RTX; BV	Relapse, PD
14	F	13 / 13	Undefined / CID	NHL / T-cell lymphoma	4A	Submandibular	-/-	POG 9404	None	CR
15	M	15 / 14	GIMAP5 / CID	HL / nodular lymphocyte-predominant subtype	3A	Axillary	-/-	ABVD	RTX	CR
16	F	25 / 23	GIMAP5 / CID	HL / nodular lymphocyte-predominant subtype	3A	Axillary	-/-	ABVD → ICE	RTX	Relapse

17	M	12 / 11	Undefined / CID	NHL / Burkitt lymphoma	4A	Colon	-/-	POG 9317 → ICE	RTX	PD
18	F	3 / 18	Ataxia-telangiectasia / DNA repair defect	Solid / CNS tumor	NA	Intracranial	-/-	Doxorubicin	None	PD
19	M	6 / 5	MAGT1 / ID	NHL / DLBCL	3A	Cervical	-/-	CHOP → ICE → gemcitabine + oxaliplatin + lenalidomide → vincristine + obinutuzumab + bendamustine + ibrutinib	RTX; nivolumab	Relapse, PD
20	F	6 / 6	Undefined / CID	NHL / Burkitt lymphoma	4A	Ovarian	+/-	COG ANHLO1P1	RTX	CR
21	F	18 / 11	GIMAP5 / CID	HL / classical HL	NA	NA	NA/NA	NA	NA	Relapse
22	F	19 / 19	GIMAP5 / CID	HL / nodular lymphocyte-predominant subtype	4A	Inguinal	-/-	ABVD	RTX	CR
23	F	32 / 32	ADA2 / AID	NHL / T-cell lymphoma	4B	Axillary	+/-	CHOP	RTX	PD
24	F	18 / 15	CVID / PAD	HL / classical HL	4A	Cervical	-/-	ABVD	None	Relapse
25	M	9 / 9	CD137 / ID	NHL / DLBCL	4B	Cervical	+/+	CHOP → BFM-95	RTX	PD
26	M	18 / 17	CVID / PAD	HL / mixed cellularity subtype	3B	Axillary	+/+	ABV-COPP	None	CR

27	M	13 / 12	BCAP31 / CID	HL / mixed cellularity subtype	4B	Cervical	+/+	ABVD	RTX	CR
28	M	1 / 2	Undefined / CID	NHL / aggressive B-cell lymphoma	4B	Colon	+/+	None	RTX	PD
29	M	39 / 46	CVID / PAD	Solid / Adenocarcinoma	NA	Colon	-/-	NA	NA	PD
30	M	7 / 5	CVID / PAD	NHL / Burkitt lymphoma	2E	Ileum	-/-	COPAD LMB-96	None	CR
31	F	4 / 4	CVID / PAD	NHL / Burkitt lymphoma	4B	Orbit	-/+	COG	RTX	CR
32	F	1 / 7	DOCK8 / CID	NHL / Aggressive B-cell lymphoma	3A	Intracranial	NA/NA	POG 9317	RTX	NA
33	M	12 / 13	DCLRE1C / CID	HL / nodular sclerosis subtype†	2A	Submandibular	NA/NA	ABVD → ABVD	None	Relapse
34	F	12 / 11	STK4 / CID	NHL / lymphoplasmacytic lymphoma	4A	Submandibular	+/+	LMB-96 → ABVD	RTX	Relapse
35	F	3 / 3	DOCK8 / CID	NHL / Aggressive B-cell lymphoma	4A	Intracranial	NA/NA	POG 9317	NA	PD
36	M	2 / 10	Ataxia-telangiectasia	NHL / T-cell lymphoma	3A	NA	NA/NA	BFM non-B	None	CR

			/ DNA repair defect							
37	M	4 / 4	ALPS / ID	NHL / T-cell lymphoma	4A	Abdomen	-/+	BFM non-B	None	PD
38	F	7 / 12	Ataxia-telangiectasia / DNA repair defect	NHL / T-cell-rich large B-cell lymphoma	4A	NA	NA/NA	BFM non-B	None	PD
39	F	3 / 9	Ataxia-telangiectasia / DNA repair defect	NHL / B-cell lymphoblastic lymphoma	3A	NA	NA/NA	LMB-96 → CHOP	RTX	PD
40	F	4 / 5	Ataxia-telangiectasia / DNA repair defect	NHL / DLBCL	2B	Lung	+/+	BFM-95R3 → EPOCH	RTX	Relapse, PR
41	F	4 / 5	Ataxia-telangiectasia / DNA repair defect	NHL / DLBCL	2E	Orbit	NA/NA	POG 9317	None	PD
42	F	2 / 10	Ataxia-telangiectasia / DNA repair defect	NHL / aggressive B-cell lymphoma	NA	NA	NA/NA	POG 9317	NA	PD
43	M	9 / 15	WAS / CID	NHL / aggressive B-cell lymphoma	3A	Nasopharynx	-/-	POG 9317	None	Relapse
44	F	12 / 12	CVID / PAD	HL / classical HL	3A	Axillary	-/-	COPP → ABVD	RTX	CR

45	M	6 / 7	Ataxia-telangiectasia / DNA repair defect	HL / nodular sclerosis subtype	2A	Testicular	+/NA	ABVD	None	CR
46	M	3 / 9	Ataxia-telangiectasia / DNA repair defect	HL / classical HL	NA	NA	NA/NA	ABVD	None	CR
47	M	4 / 4	XLP / ID	HL / classical HL	NA	NA	NA/NA	COPP	None	CR
48	F	4 / 4	ITK / CID	HL / mixed cellularity subtype	3B	Lung	+/+	ABVD	RTX	CR
49	M	5 / 1	Nijmegen breakage syndrome / DNA repair defect	Solid / Rhabdomyosarcoma	NA	Anal	NA/NA	NA	None	Relapse
50	F	32 / 30	PIK3CD / CID	HL / classical HL	4A	Lung	-/+	ABVD → ICE → Br-GDP	RTX; BV; nivolumab; favezelimab	Relapse, PD
51	M	11 / 11	TTP2 deficiency / ID	HL / nodular sclerosis subtype	4B	Supraclavicular	+/+	ABVD	None	CR
52	M	13 / 12	Undefined / CID	NHL / Burkitt lymphoma	3A	Spleen	NA/+	BFM 2012	None	CR
53	F	6 / 6	NFKB1 / PAD	Solid / Glioblastoma	NA	Intracranial	-/-	None	None	PD
54	M	11 / 11	Undefined / ID	HL / nodular sclerosis subtype	4B	Supraclavicular	+/+	ABVD → RT (18 doses)	RTX; BV	Relapse

55	M	6 / 6	Undefined / ID	NHL / Burkitt lymphoma	4A	Colon	+/+	POG 9317	RTX	CR
56	F	6 / 6	Undefined / CID	NHL / DLBCL	4B	Cervical	+/+	None	None	PD
57	M	3 / 3	CVID / PAD	NHL / Burkitt lymphoma	4A	Peritoneum	-/-	COG ANHLO1P1	RTX	CR
58	M	2.5 mo / 4 mo	IL-2R deficiency / CID	NHL / DLBCL	1E	Lumbar vertebra	-/-	None	None	CR
59	M	4 / 4	Undefined / ID	NHL / Burkitt lymphoma	2E	Peritoneum	-/+	COPAD	RTX	CR
60	F	16 / 16	CVID / PAD	NHL / Burkitt lymphoma	4B	Maxillary	-/-	BFM 2012	None	Relapse
61	M	13 / 12	Undefined / CID	HL / mixed cellularity subtype	4A	Supraclavicu lar	-/-	ABVD	None	CR
62	M	4 / 9	CVID / PAD	HL / nodular sclerosis subtype	3A	Cervical	-/-	ABVD → Vinorelbine + Gemcitabine	RTX; BV	Relapse
63	F	11 / 11	CVID / PAD	NHL / T-cell lymphoma	3A	Axillary	-/-	POG 9404	RTX	PD
64	M	14 / 14	Undefined / CID	HL / nodular sclerosis subtype	3A	Cervical	+/+	ABVD → RT (18 doses)	None	CR
65	M	5 mo / 8	Partial DiGeorge syndrome / CID	Solid / pilocytic astrocytoma	NA	Orbit	-/-	Gross total resection	None	CR
66	M	3 mo / 5	DOCK8 / CID	plasmacytom a	NA	Colon	-/-	None	None	CR
67	M	6 mo / 14	DOCK8 / CID	plasmacytom a	NA	Cervical	-/-	None	None	CR

Table S1 (continued).

Patient	HSCT	Follow-up (months)	Outcome
1	yes	127	Alive
2	no	1	Deceased
3	no	3	Deceased
4	yes	52	Deceased
5	no	144	Alive
6	yes	92	Alive
7	no	95	Alive
8	yes	87	Alive
9	no	57	Alive
10	yes	273	Alive
11	yes	174	Alive
12	no	86	Alive
13	no	43	Deceased
14	no	73	Alive
15	no	61	Alive
16	no	83	Alive
17	no	19	Deceased
18	no	8	Deceased
19	no	21	Deceased
20	no	4	Alive

21	no	153	Deceased
22	no	46	Alive
23	no	2	Deceased
24	no	66	Alive
25	no	24	Deceased
26	no	91	Alive
27	no	14	Alive
28	no	0	Deceased
29	no	2	Deceased
30	no	79	Alive
31	no	61	Alive
32	no	155	Deceased
33	yes	77	Alive
34	no	164	Alive
35	no	11	Deceased
36	no	6	Discontinued follow-up
37	no	9	Deceased
38	no	2	Deceased
39	no	66	Deceased
40	no	166	Deceased
41	no	4	Deceased
42	no	6	Deceased
43	no	62	Deceased
44	no	273	Alive
45	no	161	Alive
46	no	78	Alive
47	no	168	Alive
48	yes	143	Alive
49	no	52	Deceased
50	no	110	Deceased
51	no	19	Alive

52	yes	50	Alive
53	no	0	Deceased
54	no	18	Alive
55	no	87	Alive
56	no	0	Deceased
57	no	31	Alive
58	yes	25	Alive
59	no	14	Alive
60	no	10	Deceased
61	no	10	Alive
62	no	60	Alive
63	no	84	Alive
64	no	180	Alive
65	no	190	Alive
66	yes	160	Alive
67	yes	141	Alive

Abbreviations: ABVD, doxorubicin, bleomycin, vinblastine, dacarbazine; ABV-COPP, doxorubicin, bleomycin, vinblastine, cyclophosphamide, vincristine, procarbazine, prednisone/prednisolone; AID, autoinflammatory disease; ALPS, autoimmune lymphoproliferative syndrome; BFM, Berlin-Frankfurt-Munster; Br-GDP, brentuximab vedotin, gemcitabine, dexamethasone, cisplatin; BV, brentuximab vedotin; CHOP, cyclophosphamide, doxorubicin, vincristine, prednisone/prednisolone; CID, combined immunodeficiency; COG, Children's Oncology Group; COPAD, cyclophosphamide, vincristine, prednisone/prednisolone, doxorubicin; COPP, cyclophosphamide, vincristine, procarbazine, prednisone/prednisolone; CR, complete response; CVID, common variable immunodeficiency; DLBCL, diffuse large B-cell lymphoma; DOCK8, dedicator of cytokinesis 8; EBER, Epstein-Barr virus-encoded RNA; EBV, Epstein-Barr virus; GDP, gemcitabine, dexamethasone, cisplatin; HL, Hodgkin lymphoma; HSCT, hematopoietic stem cell transplantation; ICE, ifosfamide, carboplatin, etoposide; ID, immune dysregulation; IEI, inborn error of immunity; NA, not available; NHL, non-Hodgkin lymphoma; PAD, predominantly antibody deficiency; PD, progressive disease; PR, partial response; RT, radiotherapy; RTX, rituximab; WAS, Wiskott-Aldrich syndrome; XLP, X-linked lymphoproliferative disease. Ages are presented in years unless otherwise specified. EBV status is presented as serum PCR / tissue EBER. * Relapse malignancy: NHL / DLBCL. † Relapse malignancy: HL / mixed cellularity lymphoma.

Table S2. Univariable and Clinically Selected Multivariable Cox Regression Analyses of Factors Associated with Overall Survival

Variable	Univariable HR (95% CI)	p value	Multivariable HR (95% CI)	p value
**Malignancy type**		**<0.001**		**0.001**
**HL**	Reference		Reference	
**NHL**	7.16 (2.06-24.81)	0.002	7.43 (2.15-25.71)	0.002
**Solid/plasma cell tumor**	7.80 (1.70-35.85)	0.008	6.12 (1.29-29.06)	0.023
**Age at malignancy diagnosis, per year**	1.04 (0.98-1.10)	0.210	1.07 (1.01-1.13)	0.016
**Age at IEI diagnosis, per year**	1.00 (0.94-1.06)	0.971	Not included	—
**DNA repair defect**				
**Other IEI groups**	Reference		Reference	
**DNA repair defect**	**2.72 (1.18-6.29)**	**0.019**	**2.73 (1.10-6.80)**	**0.031**

Notes: Variables in the multivariable model were selected by considering univariable findings together with clinical relevance and previously reported prognostic importance; no fixed univariable p-value threshold was used as the sole criterion for model entry; the model included malignancy type, age at malignancy diagnosis, and DNA repair defect status. Plasma cell tumors were combined with solid tumors because the plasma cell tumor category included only two patients and no deaths, leading to sparse-data/separation problems in the four-category Cox model. The multivariable model included 67 patients and 26 deaths. The proportional hazards assumption was not violated (global test p = 0.30). Age at IEI diagnosis was retained in the univariable analysis but was not included in the multivariable model because it was strongly correlated with age at malignancy diagnosis (Pearson r = 0.83; Spearman's rho = 0.67, p < 0.001). Age at malignancy diagnosis was selected because OS follow-up began at malignancy diagnosis. The two age estimates should not be interpreted as independent effects.

Figure S1. Genetic testing strategy, variant screening, and diagnostic yield.
Retrospective genetic testing strategy in 67 patients with IEI-associated malignancies over 21 years. Testing included single-gene analysis, EBV-focused NGS panel testing, WES, and FISH. A molecular/genetic diagnosis was established in 45 patients (67.2%), whereas 22 patients (32.8%) remained undiagnosed.

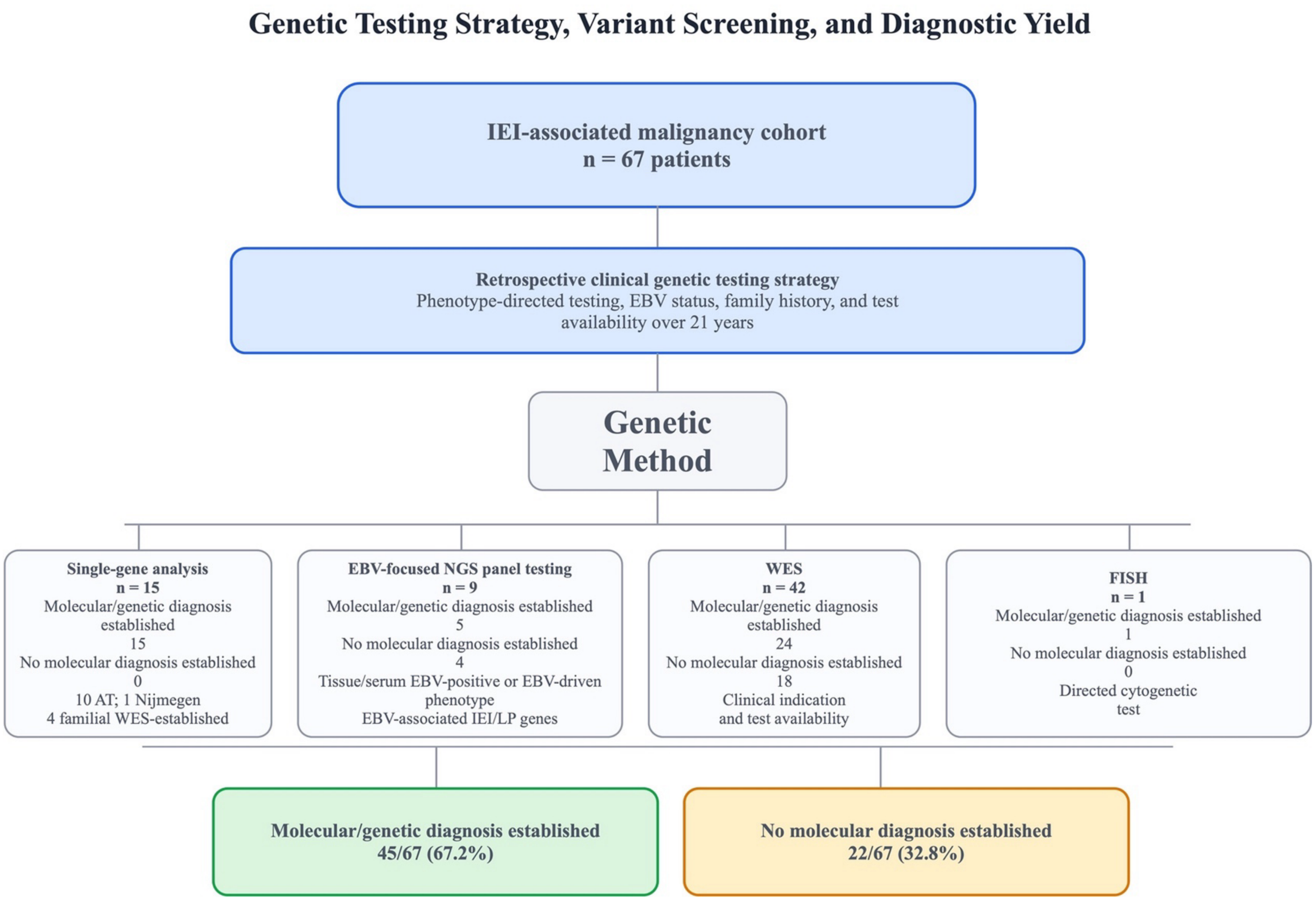

Figure S2. WES workflow and variant-screening strategy.
Standardized workflow applied to whole-exome sequencing performed from 2019 onward, including sample/library preparation, sequencing, bioinformatic analysis, variant filtering and prioritization, followed by variant interpretation and validation.

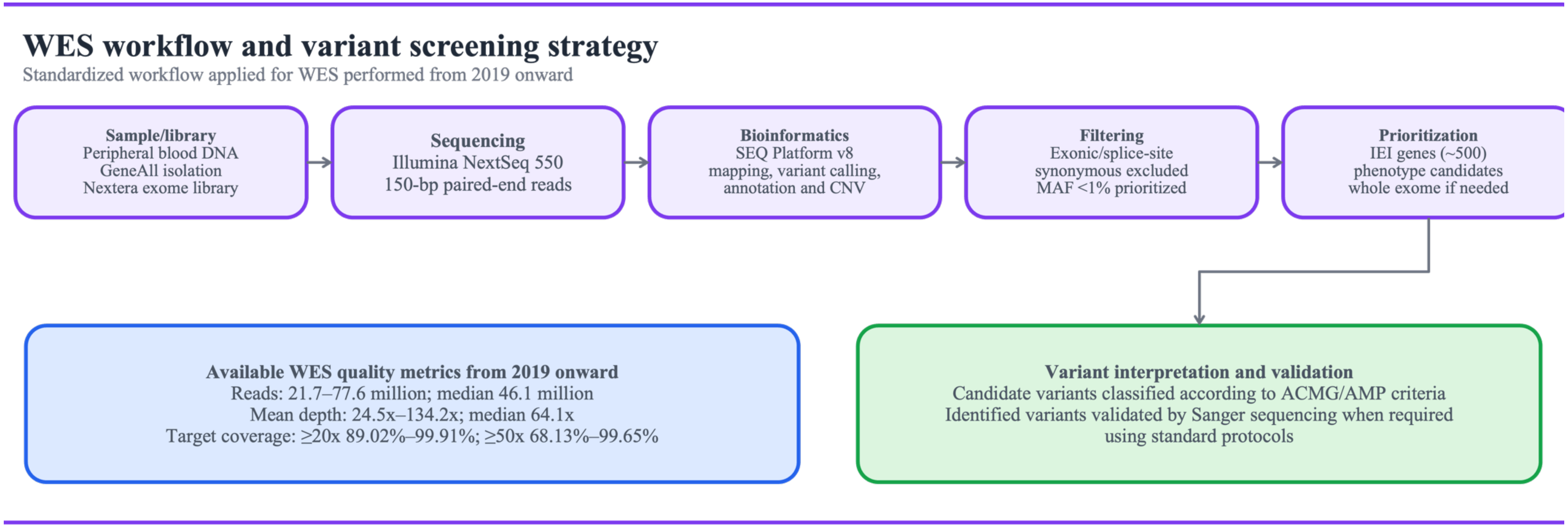

Supplement: Supplementary file 1 [file DataSheet1.pdf]
